# VEGFA Expression Is Inhibited by Arsenic Trioxide in HUVECs through the Upregulation of Ets-2 and miRNA-126

**DOI:** 10.1371/journal.pone.0135795

**Published:** 2015-08-14

**Authors:** Hong-yan Ge, Zhong-jing Han, Pei Tian, Wen-jie Sun, Da-xi Xue, Yu Bi, Zhang-hui Yang, Ping Liu

**Affiliations:** 1 Key Laboratory of Harbin Medical University Eye Center, Eye Hospital, First Affiliated Hospital, Harbin Medical University, 23 Youzheng Street, Harbin, China; 2 Daqing Oilfield General Hospital,9 ZhongKang street, Saertu District of Daqing, China; 3 Department of Biochemistry and Molecular Biology, Harbin Medical University, Harbin, China; 4 Department of Cardiology, The Second Affiliated Hospital of Harbin Medical University, Key Laboratory of Myocardial Ischemia Mechanism and Treatment (Harbin Medical University), Ministry of Education, Baojian Road, Harbin, Heilongjiang Province, PR China; INSERM-Université Paris-Sud, FRANCE

## Abstract

Arsenic trioxide (ATO) has been used to treat patients with acute promyelocytic leukemia. Recently, studies have shown that ATO can induce apoptosis in leukemic cells and blood vessel endothelial cells in a time- and dose-dependent manner through the inhibition of vascular endothelial growth factor A (VEGFA) production. VEGFA is a key factor in angiogenesis initiation. Targeted inhibition of VEGF or VEGFA expression can suppress angiogenesis; however, little is known about the mechanism by which ATO inhibits VEGFA expression. In this study, we investigated the role of miRNA-126 in the mechanism of action of ATO in human umbilical vein endothelial cells (HUVECs). ATO significantly decreased the viability and proliferation of HUVECs and decreased their migration at 48 h. Cell proliferation was inhibited by 50% (IC50) when 5.0 μmol/L ATO was used. ATO treatment induced miR-126 upregulation and HUVEC apoptosis. Transfection with a miR-126 mimic significantly downregulated VEGFA mRNA levels, and transfection with a miR-126 inhibitor significantly upregulated VEGFA mRNA levels. Finally, we showed that ATO treatment upregulated Ets-2 and miR-126 expression in HUVECs. These results demonstrate that ATO inhibits the growth of HUVECs and induces apoptosis by downregulating VEGFA. One mechanism by which this occurs is Ets-2 upregulation, which results in an increase in miR-126 levels and downregulation of VEGFA expression.

## Introduction

Angiogenesis is the formation of new blood vessels during growth and development. This process occurs normally during reproduction, embryonic development, and wound repair. However, angiogenesis also occurs during disease-related processes, including tumor growth, wound healing, and the restoration of blood flow to tissues after injury [1,2,3]. Endothelial cells (ECs) play an essential role in vascular development and function, the maintenance of vascular integrity, angiogenesis, and wound repair [4]. In recent years, many clinical trials have shown that ECs are the target of angiogenesis inhibitors that can selectively influence diverse EC functions [5,6]. Vascular endothelial growth factor (VEGF) is the main regulatory factor in angiogenesis [7,8], and the targeted inhibition of VEGF or VEGFA expression can suppress this process [9].

Arsenic has been used to treat a wide variety of illnesses, including plague, malaria, and er; it is also used to promote sweating. In the 1990s, arsenic trioxide (ATO) was used to treat patients with acute promyelocytic leukemia (APL) [10,11,12,13]. Roboz et al. showed that ATO can induce apoptosis in leukemic cells and blood vessel endothelial cells in a time- and dose-dependent manner by inhibiting VEGFA production [14]. Recently, Xiao et al. demonstrated that ATO inhibited VEGFA and vascular endothelial growth factor receptor (VEGFR) expression and suppressed both angiogenesis and gastric tumor growth *in vivo* and *in vitro* [15,16]. After 48 h of exposure to intermediate doses of arsenic, cell proliferation was completely inhibited [14,17]. Consistently, arsenic has been shown to be toxic to endothelial cells in culture when the threshold of 5.0 μmol/L is exceeded, especially when the cells are in the exponential phase of growth [14]. The molecular mechanism by which ATO inhibits angiogenesis remains unclear. To date, no single mechanism that explains all of the effects observed after ATO treatment has been discovered, indicating that ATO may act at multiple levels and through several modes of action. ATO might also induce endothelial cell apoptosis and regulate growth factor responses through the regulation of microRNA (miRNA) expression.

miRNAs are single-stranded 20–25-nucleotide RNA molecules that post-transcriptionally regulate the expression of their target mRNAs either by translational inhibition or by destabilizing the target mRNAs [18,19]. Increasing evidence indicates that miRNAs play important roles in the response of the cardiovascular system to injury and stress [20,21]. A recent study has demonstrated that miR-126 is located in an intron of EGFL-7, a gene that is highly expressed in endothelial cells [22]. Fish et al. utilized microRNA profiling of endothelial cells derived from embryonic stem (ES) cells to identify a group of endothelial-enriched microRNAs, including miR-126, miR-146, miR-197, and miR-625. The authors found that miR-126 negatively regulates VEGFA signaling in endothelial cells both *in vivo* and *in vitro* and showed that miR-126 directly regulates PI3KR2 (p85-b) and SPRED1, both of which are negative regulators of the VEGFA signaling pathway [23].

The E26 transformation-specific sequence (Ets) factors belong to a family of transcription factors that share a highly conserved DNA-binding domain and regulate cell development, senescence, and death in addition to tumorigenesis [24,25,26]. Several members of the Ets family are expressed in endothelial cells and have roles in vasculogenesis, angiogenesis, inflammation, and remodeling [24,26,27]. Several studies have shown that the Ets family members Ets-1 and Ets-2 induce the expression of miR-126 in endothelial cells [28].

We therefore decided to investigate the inhibitory effect of ATO on human umbilical vein endothelial cell (HUVEC) proliferation, migration, and apoptosis as well as its effect on the expression of miR-126 and Ets-2.

## Materials and Methods

### Cell lines

HUVECs (AllCells, Shanghai, China) were cultured in complete medium (HUVEC-004, supplied by AllCells) in an atmosphere containing 5% CO_2_ at 37°C. All experiments were performed according to the manufacturer’s instructions.

### MTT assay

The inhibitory effect of ATO on HUVEC growth was analyzed using an MTT assay. Briefly, cells (5,000/well) were plated in 96-well plates in complete medium (HUVEC-004) and incubated for 48 h in the presence of various concentrations of ATO (0, 1.0, 2.5, 5.0, 7.5, 10.0, 12.5, 15.0, 17.5, and 20.0 μmol/L). MTT was added to a final concentration of 0.45 mg/ml, and the cells were incubated for a further 4 h at 37°C. The medium was then removed, and 150 μl of dimethyl sulfoxide (DMSO) was added for 30 min at room temperature to dissolve the blue formazan crystals. The spectrophotometric absorbance of the samples at 492 nm was measured using a microplate reader. The percentage of viable cells was calculated as follows: (A_492_ of the experimental group/A_492_ of the control group) × 100.

### Scratch wound assay

HUVEC migration was detected using a scratch wound assay. The cell monolayer was scraped with a sterile cell scraper to create a cell-free zone. The cells were washed with medium and stimulated with 2.5 μmol/L ATO as indicated. Using an inverted microscope, we recorded endothelial cell migration at 5 distinct positions at the time of injury and after 48 h of cultivation.

### Oligonucleotide design and synthesis

The sequences of mature miRNA-126 are available at the miRNA Registry. The miR-126 mimic and miR-126 inhibitor oligonucleotides were designed using sequences that were complementary to mature miRNA-126. The oligodeoxynucleotide sequences used in this study were as follows: Hsa-miR-126 mimic sense: 5’-UCG UAC CGU GAG UAA UAA UGC G-3’; Hsa-miR-126 mimic antisense: 5’-CAU UAU UAC UCA CGG UAC GAU U-3’; Hsa-miR-126 inhibitor: 5’-CGC AUU AUU ACU CAC GGU ACG A-3’; and miRNA inhibitor NC: 5’-CAG UAC UUU UGU GUA GUA CAA-3’. All the oligodeoxynucleotides were chemically synthesized and modified with phosphorothioate (Shanghai GenePharma, Shanghai, China). miR-126-3p (Accession: MIMAT0000445, mirBase)

The Ets-1 sense (5'-GUG GUU UCC AGU CCA AUU ATT-3'), Ets-1 antisense (5'-UAA UUG GAC UGG AAA CCA CTT-3'), the Ets-2 sense (5'-GGG AAC AUC UGG AGC AAA UTT-3'), Ets-2 antisense (5'-AUU UGC UCC AGA UGU UCC CTT-3'), negative control sense (5’-UUC UCC GAA CGU GUC ACG UTT-3’), and negative control antisense (5’-ACG UGA CAC GUU CGG AGA ATT-3’) oligodeoxynucleotides were chemically synthesized and modified with phosphorothioate (Invitrogen, Shanghai, China).ETS-1(Accession:NM_001143820.1, genBank) ETS-2(Accession:NM_005239.5, genBank)

### ATO treatment and siRNA transfection

Briefly, 6-well plates (Costar, Cambridge, MA, USA) were coated with 2 ml/well of complete medium (HUVEC-004), and 1×10^5^ HUVECs were cultured in the wells in the presence or absence of various concentrations of ATO (1.0 μmol/L, 2.5 μmol/L, and 5.0 μmol/L). Each treatment was performed in triplicate. The plates were incubated at 37°C for 48 h in an atmosphere containing 5% CO_2_ and observed using inverted phase-contrast microscopy (Nikon, Tokyo, Japan).

HUVECs in the exponential growth phase were seeded in 6-well plates (Costar, Cambridge, MA, USA) at a density of 5×10^4^ cells/ml. The cells were transfected with miR-126 mimic, miR-126 inhibitor, and Ets-2 siRNA (oligodeoxynucleotides) using the X-tremeGENE siRNA Transfection Reagent (Roche, 68298, Mannheim, Germany) at a 5:1 volume/mass ratio of reagent to oligodeoxynucleotide in serum-free M199 for 6 h. After transfection, the cells were incubated in complete medium (HUVEC-004). Transfection of the Hsa-miR-126 mimic, the negative control, the Hsa-miR-126 inhibitor, and the miRNA inhibitor NC was performed using 1.0 μg of DNA per transfection. All transfections were performed according to the manufacturer’s instructions.

After transfection with the miR-126 mimic or the miR-126 inhibitor, either 5.0 μmol/L ATO or medium alone was added, and the transfected cells were cultured for an additional 48 h.

### Western blotting

Cells were lysed in RIPA buffer containing proteinase inhibitor (Biocolor Biosciences & Technology Company, Shanghai, China). The protein concentration of the samples was determined using BCA (Bioss, Beijing, China). Thirty micrograms of protein was separated on a 10% SDS-PAGE gel and transferred to a nitrocellulose membrane. The membranes were probed with a rabbit polyclonal primary antibody against VEGFA (Cell Biotech, Tianjin, China) and a rabbit polyclonal primary antibody against Ets-2 (Santa Cruz Biotechnology, Santa Cruz, CA, USA) at 4°C overnight, washed extensively with 0.1% Tween-20 in PBS, and incubated with secondary antibodies (1:10,000) conjugated with horseradish peroxidase. The signals were visualized using a UVP imaging system.

### RNA extraction, cDNA synthesis, and real-time PCR

The levels of miR-126, VEGFA, and Ets-2 mRNAs were measured in the HUVECs at 48 h after the treatments. Total RNA was extracted using Trizol Reagent (Invitrogen, USA) according to the manufacturer’s protocol. RNA was diluted and reverse-transcribed using the High Capacity cDNA Kit (Applied Biosystems, Foster City, CA, USA). cDNA was prepared from 1 μg of RNA from each sample via RT-PCR with random primers or with miR-126 and U6 primers. The RT-PCR products were then amplified using the TaqMan microRNA kit (Applied Biosystems, Foster City, CA, USA).

The levels of VEGFA, Ets-2, and GAPDH mRNAs were determined using the SYBR Green Real-time PCR Quantitation Kit (Invitrogen, USA) using GAPDH as an internal reference. We used the same kit to determine the expression levels of miR-126 mRNA and U6 snRNA, using U6 as an internal reference. The PCR reaction conditions were as follows: denaturation at 94°C for 3 min, followed by 45 cycles of denaturation at 94°C for 30 s, annealing at 60°C for 30 s, and synthesis at 72°C for 35 s. Fold changes in the expression levels of miR-126, VEGFA, and Ets-2 mRNA were calculated using △CT and 2^-△△CT^. All assays were performed according to the manufacturer’s instructions.

### Flow cytometry analysis

Apoptosis was measured using an Annexin V/propidium iodide (PI) flow cytometric assay (BD Biosciences, San Jose, CA, USA) according to the manufacturer’s instructions. Briefly, HUVECs were harvested, washed twice with cold PBS, and resuspended in 100 μl of binding buffer at a concentration of 1 × 10^6^ cells/ml. The cells were transferred to 5 ml culture tubes, and 5 μl of Annexin V-FITC and 10 μl of PI were subsequently added. The cells were gently vortexed and incubated in the dark for 15 min at room temperature. After 400 μL of PBS was added, the samples were analyzed on a FACScan flow cytometer (BD Biosciences, USA). Early apoptosis was estimated based on the relative number of Annexin V-FITC^+^PI^−^cells present. All experiments were performed in triplicate.

### Scanning and transmission electron microscopy

Scanning electron microscopy (SEM) and transmission electron microscopy (TEM) were used to compare the ultrastructures of HUVECs from the control and treatment groups. After 48 h, cells were prepared for imaging and analyzed under a JSM-6330F scanning electron microscope.

For TEM, HUVECs were cultured and treated as described for the ATO treatment and miR-126 mimic transfection experiments. Following a 48-h incubation in complete medium, monolayers were washed three times in PBS and detached from the plastic surface with trypsin. The cell slurry was then immediately centrifuged for 4 min at 15,000 x g. The cell pellet were washed twice with PBS and fixed in 2.5% glutaraldehyde in 0.1 M sodium cacodylate and 0.1 M sucrose (pH 7.4) overnight, then washed in 0.1 M sodium cacodylate, and postfixed in osmium tetraoxide (1.0%), stained en bloc with uranyl acetate (0.25%), dehydrated in graded ethanol solutions, and embedded in Epon. Thin sections were stained with lead citrate and then examed using 1200EX electron microscope (JEOL, Tokyo, Japan)

For SEM, HUVECs were incubated on glass coverslips in 6-well plates and grown for 48 h. Then, the cells were washed twice with PBS and fixed with 2.5% glutaraldehyde in 0.1 M sodium cacodylate and prepared for observation with a scanning electron microscope (JSM-6330F; JEOL) [29].

### Statistical analysis

All of the experiments were performed in triplicate. The data are expressed as means ± standard deviation and were compared using an ANOVA or t-test. A two-tailed Student’s t-test was used for comparisons of 2 independent groups. Statistical analysis was performed using the SPSS 13.0 software package. The results were considered statistically significant at p<0.05 (*p<0.05; **p<0.01; *** p<0.001).

## Results

### ATO reduces the viability and proliferation of HUVECs in a dose-dependent manner

**Fig 1:**
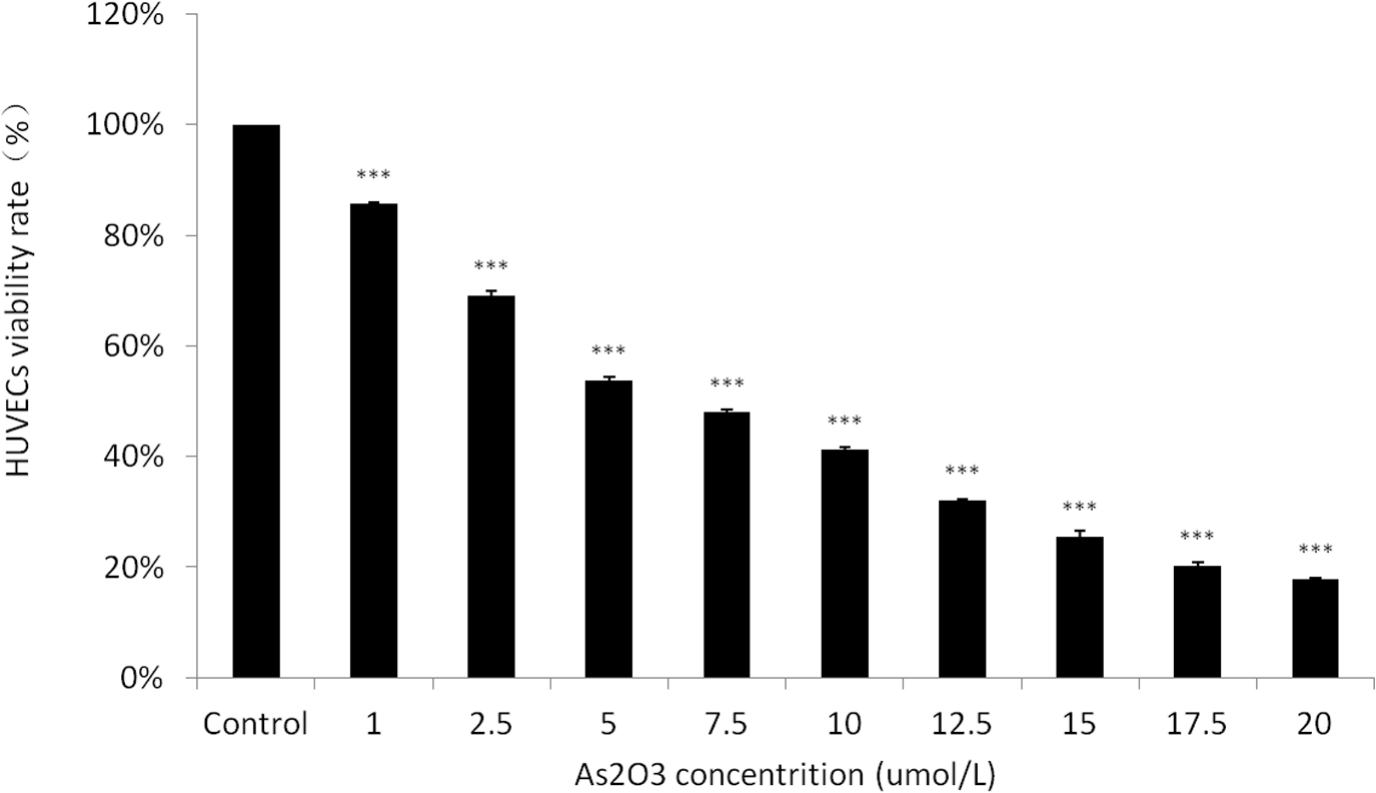
ATO dose- dependently inhibits cell viability and proliferation in HUVECs. HUVECs were plated in 96-well plates for 48 h in the presence of various concentrations of ATO (0, 1.0, 2.5, 5.0, 7.5, 10.0, 12.5, 15.0, 17.5, and 20.0 μmol/L). The effect of ATO on HUVEC growth inhibition was analyzed using an MTT assay.

To investigate the effects of ATO on the survival and proliferation of HUVECs, monolayers of rapidly proliferating cells at 70% to 80% confluence were grown for 48 h and treated with 1.0×10^−6^ to 2.0×10^−5^ mol/L ATO. Each condition was assayed in triplicate. As shown in Fig 1, the cell viability and the proliferation rates were decreased at all concentrations of ATO tested when compared with the control cells.

### ATO inhibits HUVEC migration

**Fig 2:**
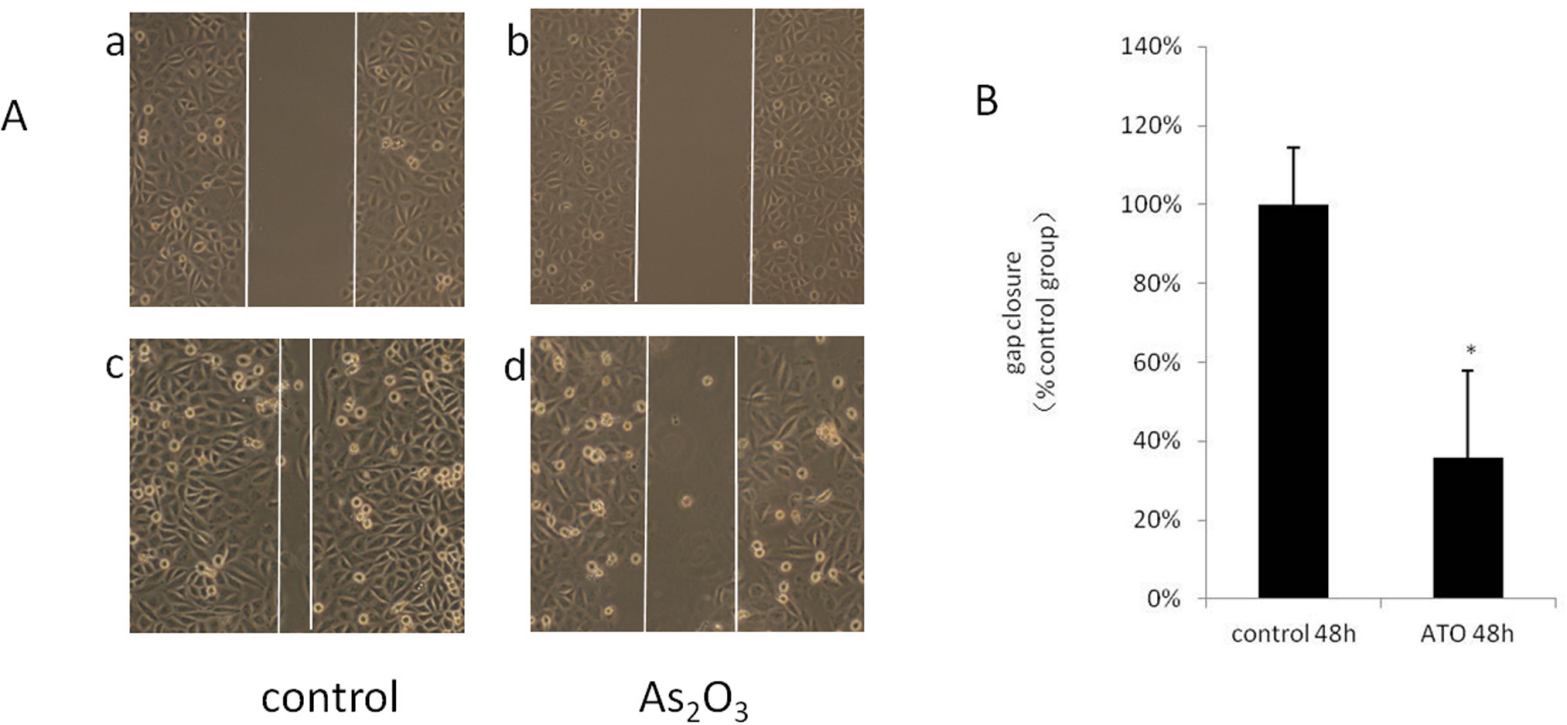
ATO inhibits migration in HUVECs. HUVEC migration was detected using a scratch wound assay. The cell monolayer was scraped with a sterile cell scraper to create a cell-free zone. The cells were washed with medium and stimulated with 5.0μmol/L ATO as indicated. Endothelial cell migration was recorded after 48 h of cultivation using an inverted microscope. (Original magnification:×100). A: a: Control group 0 hour; b: ATO 5.0 umol/L group 0 hour; c: Control group 48 h; d: ATO 5.0 umol/L group 48 h. B: Statistics result with the software of Image J showed that the rate of ATO decreased cell migration.

We studied the effect of ATO on HUVEC migration by applying ATO at a concentration of 2.5 μmol/L. At this concentration, ATO inhibited HUVEC proliferation by approximately 70%. The effect of ATO on HUVEC migration was determined by generating a “scratch” in a confluent monolayer of HUVECs and then measuring the degree of “wound closure” after 48 h in the presence or absence of ATO (2.5 μmol/L). As shown in Fig 2, ATO effectively inhibited HUVEC migration and significantly reduced the degree of wound closure compared with the control samples.

### ATO decreases VEGFA protein expression

**Fig 3:**
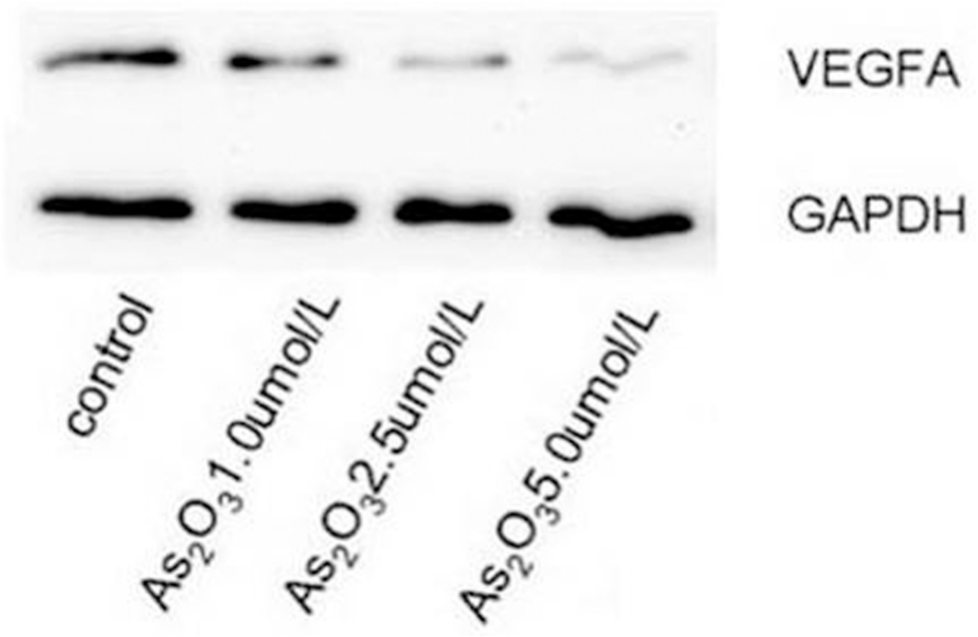
ATO decreases VEGFA protein expression. HUVECs were cultured in the presence or absence of various concentrations of ATO (1.0 μmol/L, 2.5 μmol/L, and 5.0 μmol/L). The expression of VEGFA protein was detected using the western blot assay.

To verify that the effect of ATO on VEGFA protein expression was mediated by miR-126, the cells were treated with 1.0, 2.5, and 5.0 μmol/L ATO, and VEGFA protein levels were analyzed by Western blotting. VEGFA protein levels gradually decreased as the concentration of ATO increased. Thus, ATO significantly inhibited VEGFA protein expression (Fig 3).

### ATO decreases VEGFA mRNA expression

**Fig 4:**
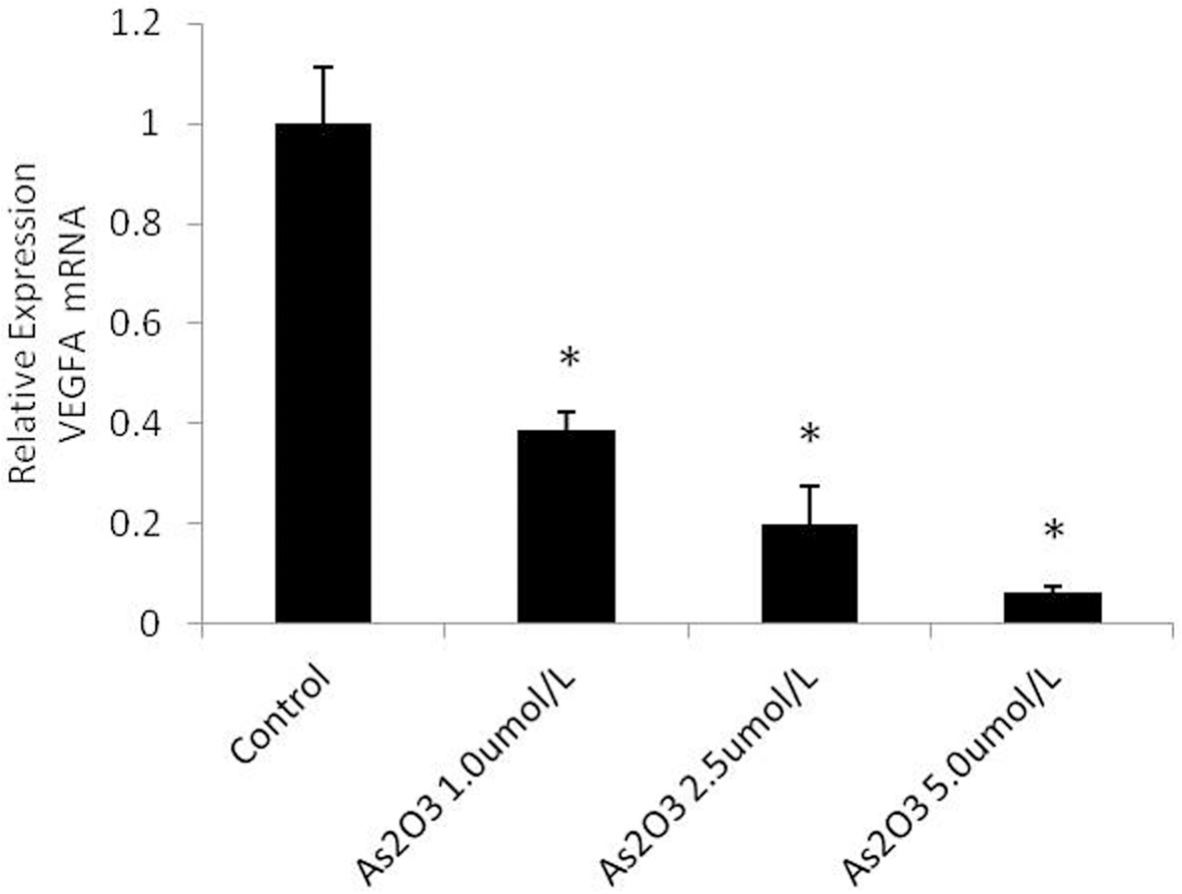
ATO decreases VEGFA mRNA expression. HUVECs were cultured in the presence or absence of various concentrations of ATO (1.0 μmol/L, 2.5 μmol/L, and 5.0 μmol/L). The expression of VEGFA mRNA was detected using the real-time PCR assay.

To determine whether the effect of ATO on VEGFA mRNA expression was mediated by miR-126, the cells were treated with 1.0 μmol/L, 2.5 μmol/L, and 5.0 μmol/L ATO, and VEGFA mRNA expression levels were analyzed using real-time PCR. We found that VEGFA mRNA expression gradually decreased with increasing concentrations of ATO (Fig 4).

### ATO upregulates miR-126 expression

**Fig 5:**
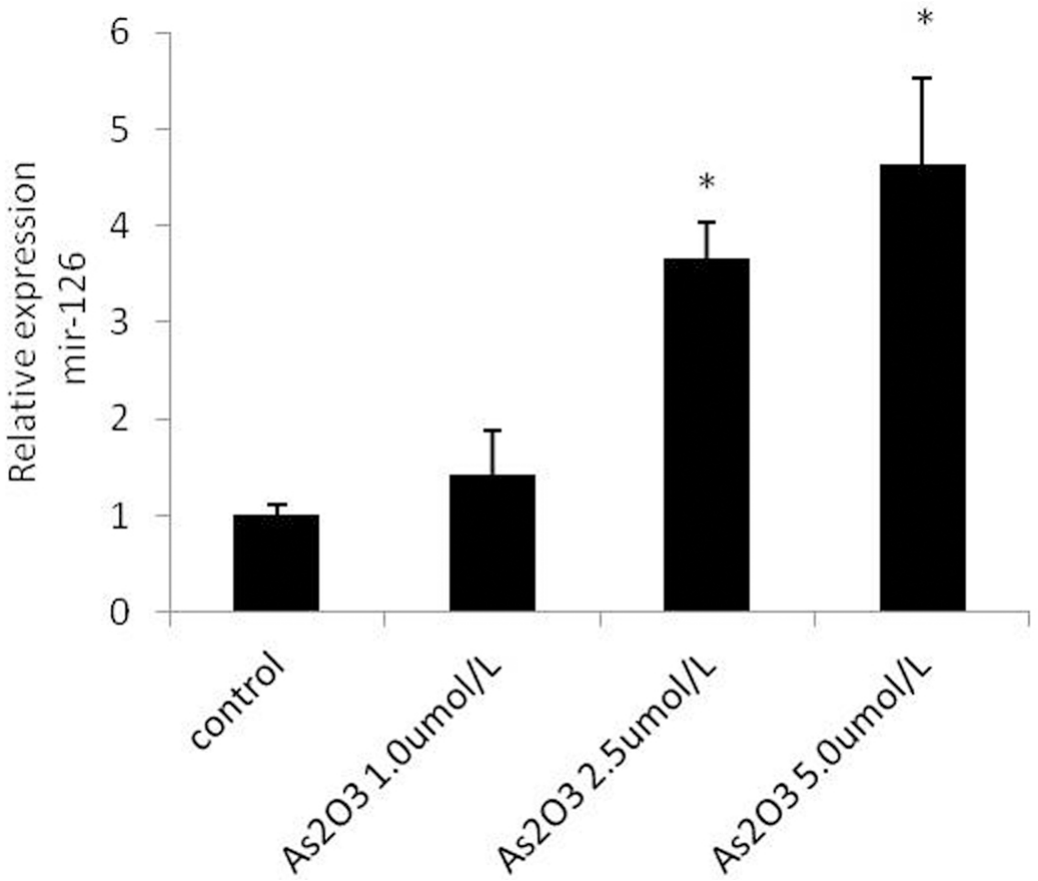
ATO upregulates miR-126 expression. HUVECs were cultured in the presence or absence of various concentrations of ATO (1.0 μmol/L, 2.5 μmol/L, and 5.0 μmol/L). The miR-126 expression levels was detected using the real-time PCR assay.

To verify that ATO dose-dependently regulated miR-126 expression in HUVECs, we treated the cells with 1.0 μmol/L, 2.5 μmol/L, and 5.0 μmol/L ATO and performed real-time PCR analysis. The resulting data showed that miR-126 expression levels gradually increased with increasing ATO concentrations (Fig 5).

### ATO, the miR-126 mimic, and the miR-126 inhibitor regulate miR-126 levels

**Fig 6:**
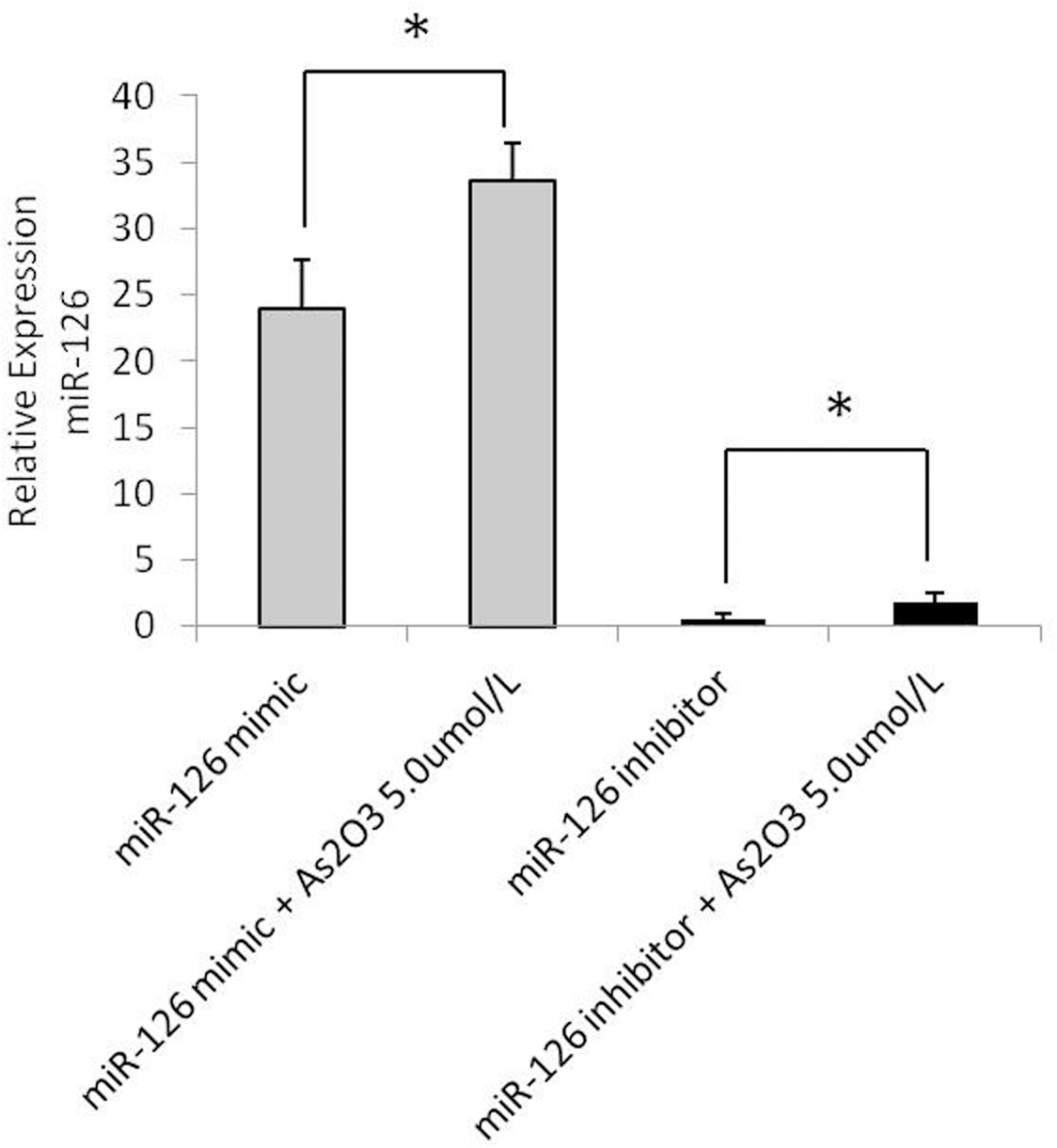
ATO and the miR-126 mimic and the miR-126 inhibitor regulate miR-126 levels. HUVECs were transfected with miR-126 mimic or miR-126 inhibitor for 6h and subsequently treated with or without high concentration (5.0umol/L) of As_2_O_3_, respectively. After transfection with the miR-126 mimic or the miR-126 inhibitor, either 5.0μmol/L ATO or medium alone was added, and the transfected cells were cultured for an additional 48h. The expression of miR-126 was detected by real-time PCR assay.

To investigate why miR-126 expression gradually increased with increasing ATO concentrations, we evaluated the effects of the miR-126 mimic and the miR-126 inhibitor on miR-126 expression levels. The miRNA levels of HUVECs transfected with the miR-126 mimic or the miR-126 inhibitor were analyzed. The results showed that the miR-126 mimic effectively upregulated miR-126, whereas the miR-126 inhibitor downregulated miR-126 (Fig 6).

We analyzed the relationship of ATO (5.0 μmol/L) with the miR-126 mimic and the miR-126 inhibitor. The results showed that ATO increased the level of miR-126. The expression of miR-126 was higher in the ATO + miR-126 mimic group than in the miR-126 mimic alone group, and it was higher in the ATO + miR-126 inhibitor group than in the miR-126 inhibitor alone group (Fig 6). These results demonstrated that ATO and the miR-126 mimic exert an additive effect on miR-126 expression upregulation.

### ATO, the miR-126 mimic, and the miR-126 inhibitor regulate VEGFA mRNA levels

**Fig 7:**
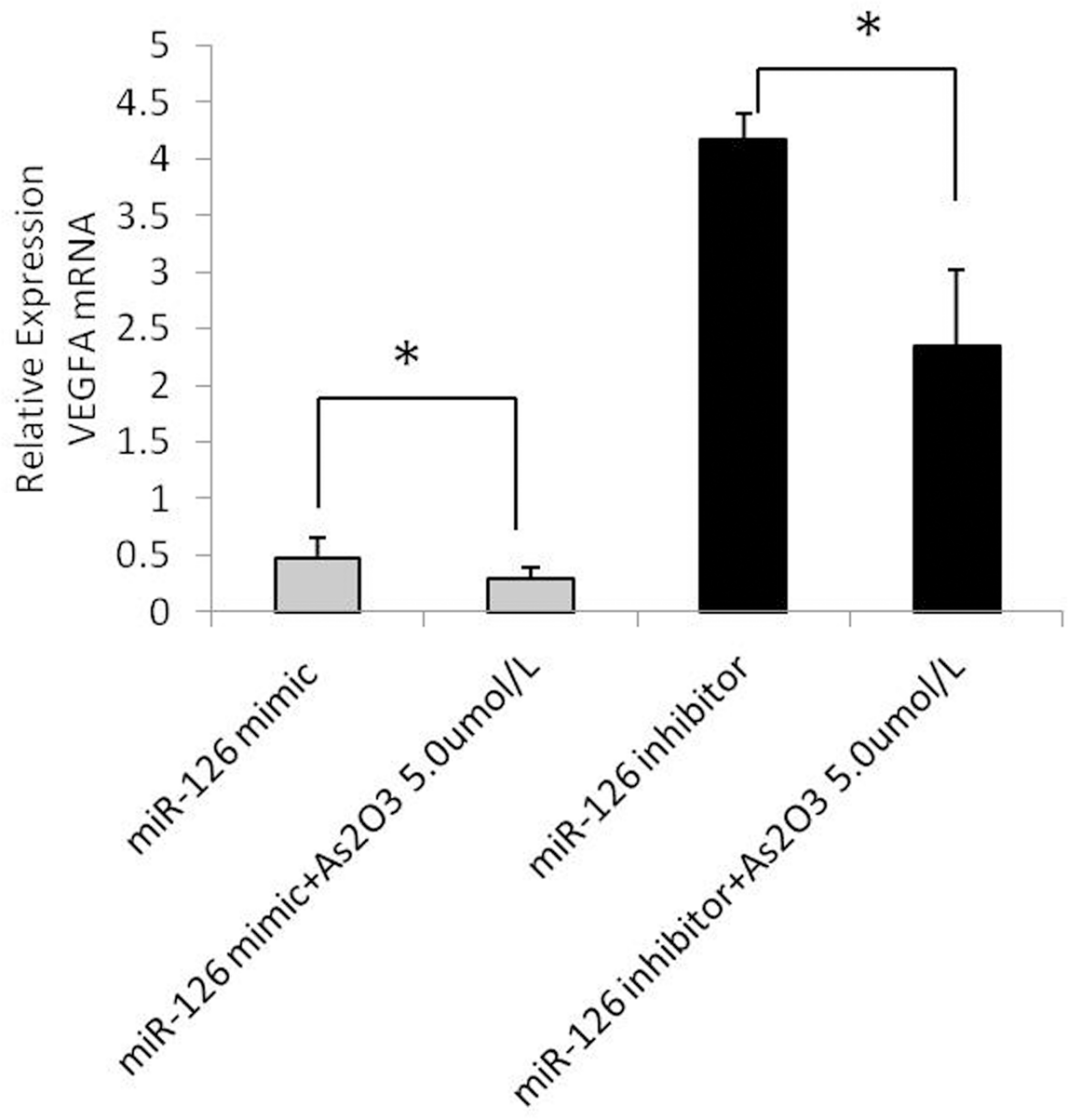
ATO and the miR-126 mimic and the miR-126 inhibitor regulate VEGFA mRNA levels. HUVECs were transfected with miR-126 mimic or miR-126 inhibitor for 6h and subsequently treated with of As_2_O_3_, respectively. After transfection with the miR-126 mimic or the miR-126 inhibitor, either 5.0 μmol/L ATO or medium alone was added, and those treated cells were cultured for an additional 48 h. The expression levels of VEGFA mRNA was detected by real-time PCR assay.

Our initial experiments indicated that ATO regulates the expression of miR-126 in HUVECs (Fig 4). To determine whether miR-126 also regulates the expression of VEGFA, we investigated the effects of the miR-126 mimic and the miR-126 inhibitor on VEGFA mRNA levels. The amount of VEGFA mRNA in the transfected HUVECs was analyzed using real-time PCR analysis (Fig 7).

The results showed that the miR-126 mimic decreased the level of VEGFA mRNA, whereas the miR-126 inhibitor increased the level of VEGFA mRNA. Moreover, the level of VEGFA mRNA expression was lower in the ATO + miR-126 mimic group than in the miR-126 mimic group, and it was lower in the ATO + miR-126 inhibitor group than in the miR-126 inhibitor group. These results demonstrated that ATO and the miR-126 mimic exerted an additive effect on the downregulation of VEGFA mRNA expression (Fig 7).

### ATO regulates the expression of Ets-1 and Ets-2 mRNA

**Fig 8:**
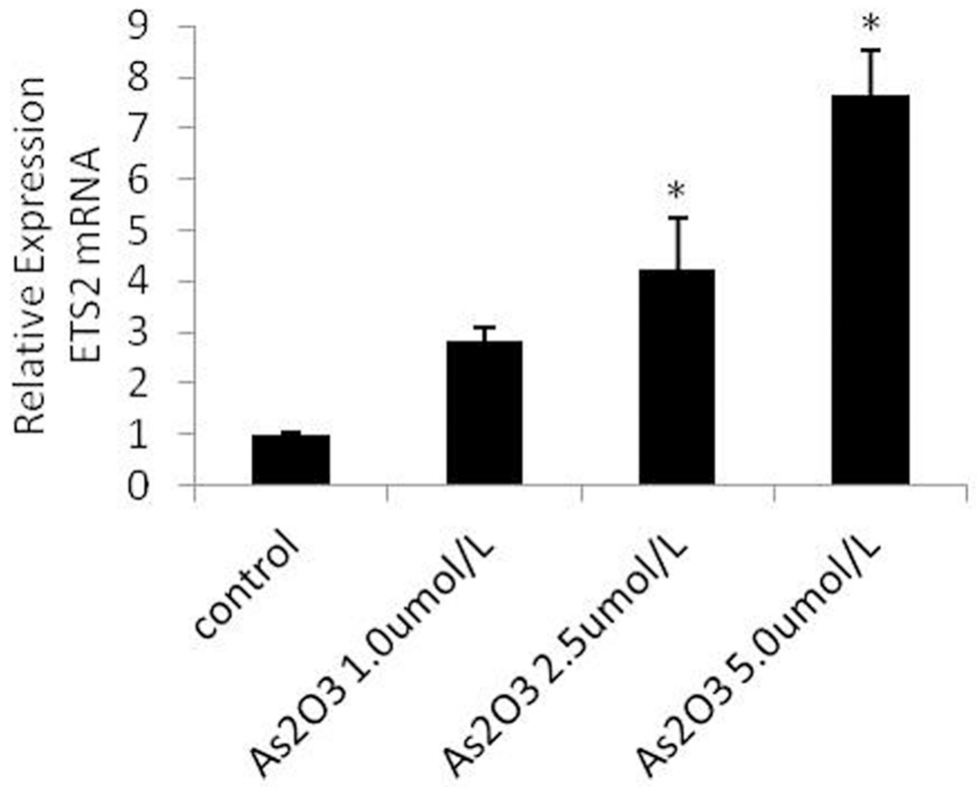
ATO regulates the expression of Ets-2 mRNA. HUVECs were cultured in the presence or absence of various concentrations of ATO (1.0 μmol/L, 2.5 μmol/L, and 5.0 μmol/L). The expression of Ets-2 mRNA was detected using the real-time PCR assay.

**Fig 9:**
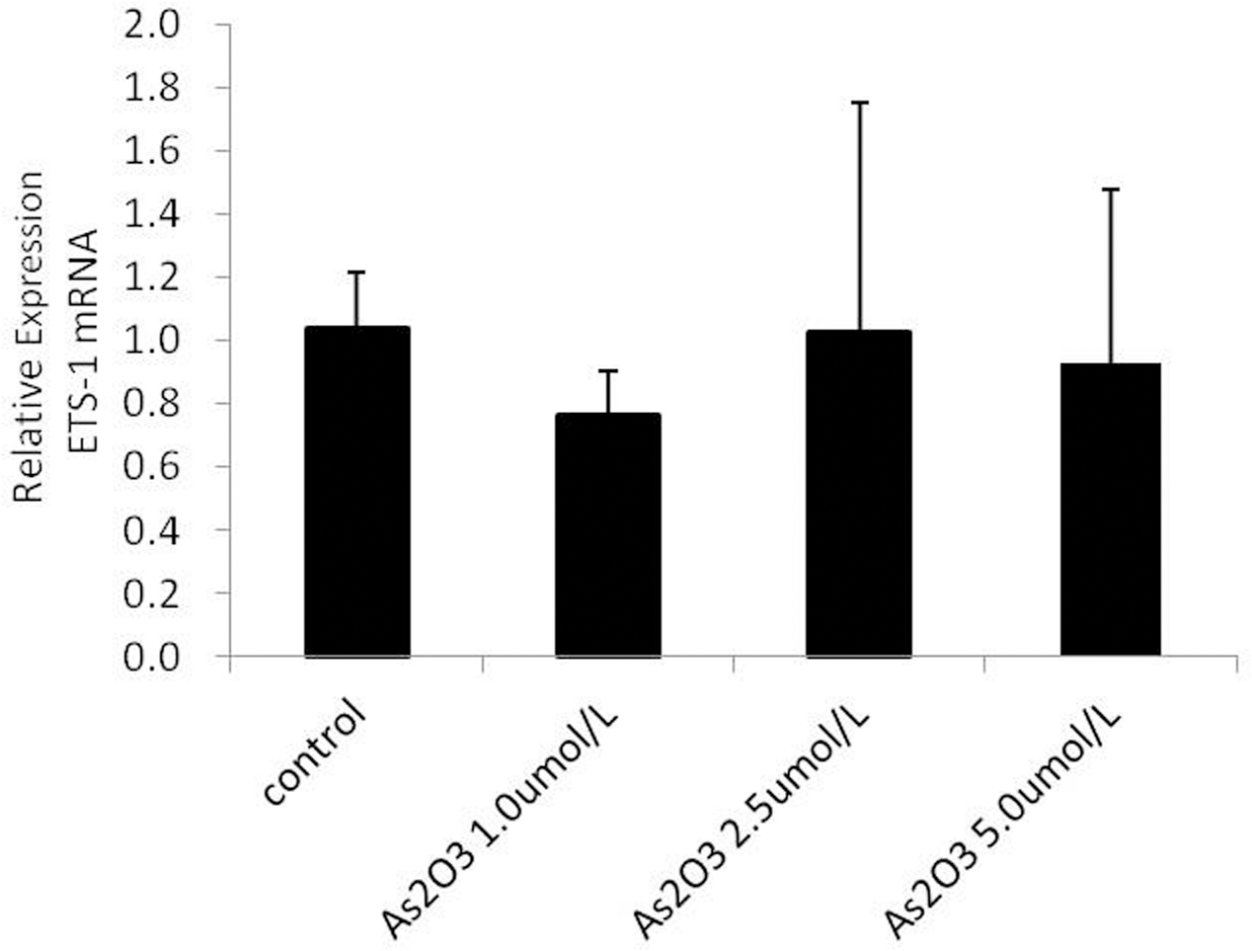
ATO regulates the expression of Ets-1. HUVECs were cultured in the presence or absence of various concentrations of ATO (1.0 μmol/L, 2.5 μmol/L, and 5.0 μmol/L). The expression of Ets-1 mRNA was detected using the real-time PCR assay.

Several studies have shown that the Ets family members Ets-1 and Ets-2 induce the expression of miR-126 in endothelial cells [28]. We hypothesized that ATO regulates miR-126 through the regulation of Ets-1 and Ets-2 mRNA. To explore this hypothesis, HUVECs were treated with 1.0 μmol/L, 2.5 μmol/L, and 5.0 μmol/L ATO and analyzed using real-time PCR. The resulting data showed that the expression of Ets-2 mRNA gradually increased at higher ATO concentrations (Fig 8); however, the Ets-1 mRNA levels did not change significantly. Thus, we did not study the relative expression of Ets-1 further (Fig 9).

### ATO regulates the expression of Ets-2 protein

**Fig 10:**
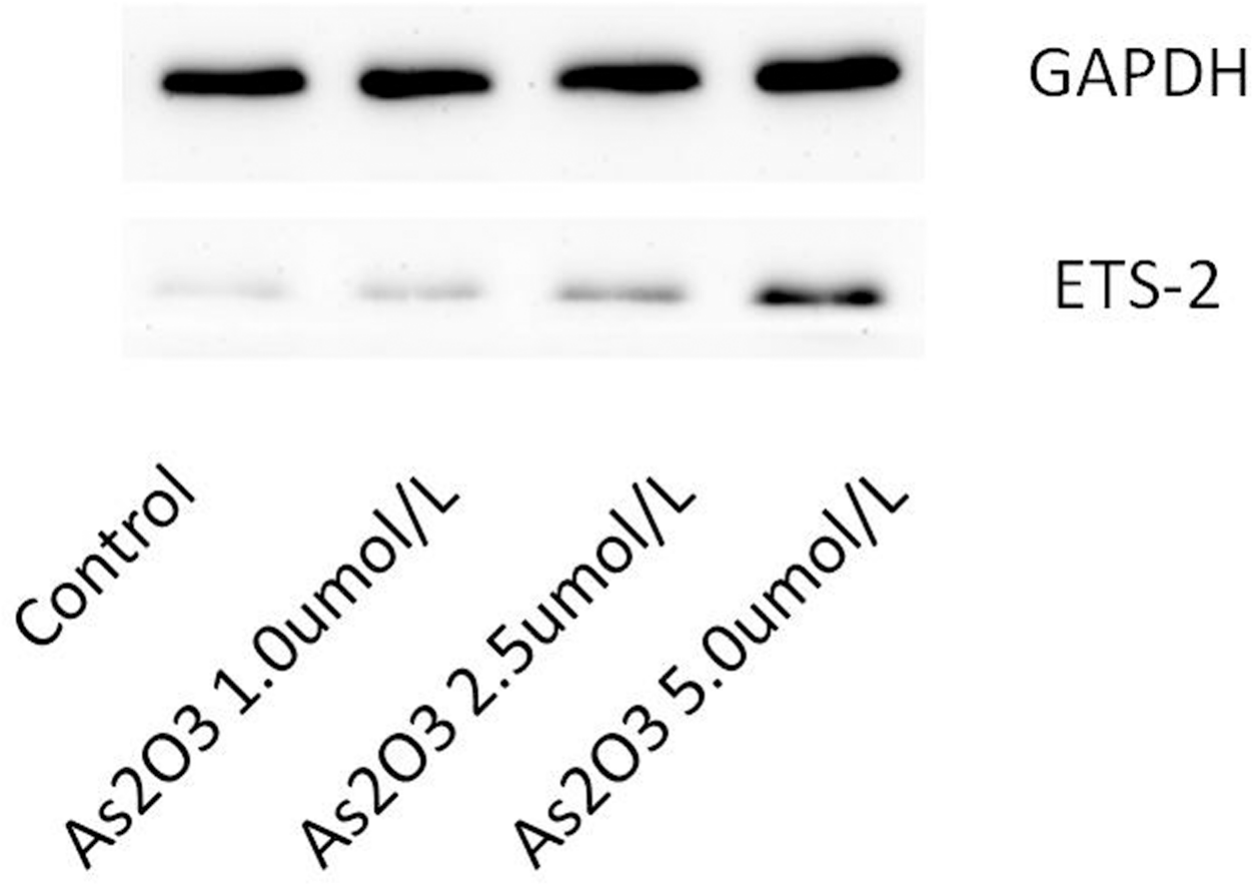
ATO regulates the expression of Ets-2 Protein. HUVECs were cultured in the presence or absence of various concentrations of ATO (1.0 μmol/L, 2.5 μmol/L, and 5.0 μmol/L). The expression of Ets-2 protein was detected using the western blot assay.

HUVECs were treated with 1.0 μmol/L, 2.5 μmol/L, and 5.0 μmol/L ATO and analyzed using western bolt. The resulting data showed that the expression of Ets-2 protein gradually increased at higher ATO concentrations (Fig 10).

### Ets-2 siRNA regulates VEGFA protein expression in HUVECs

**Fig 11:**
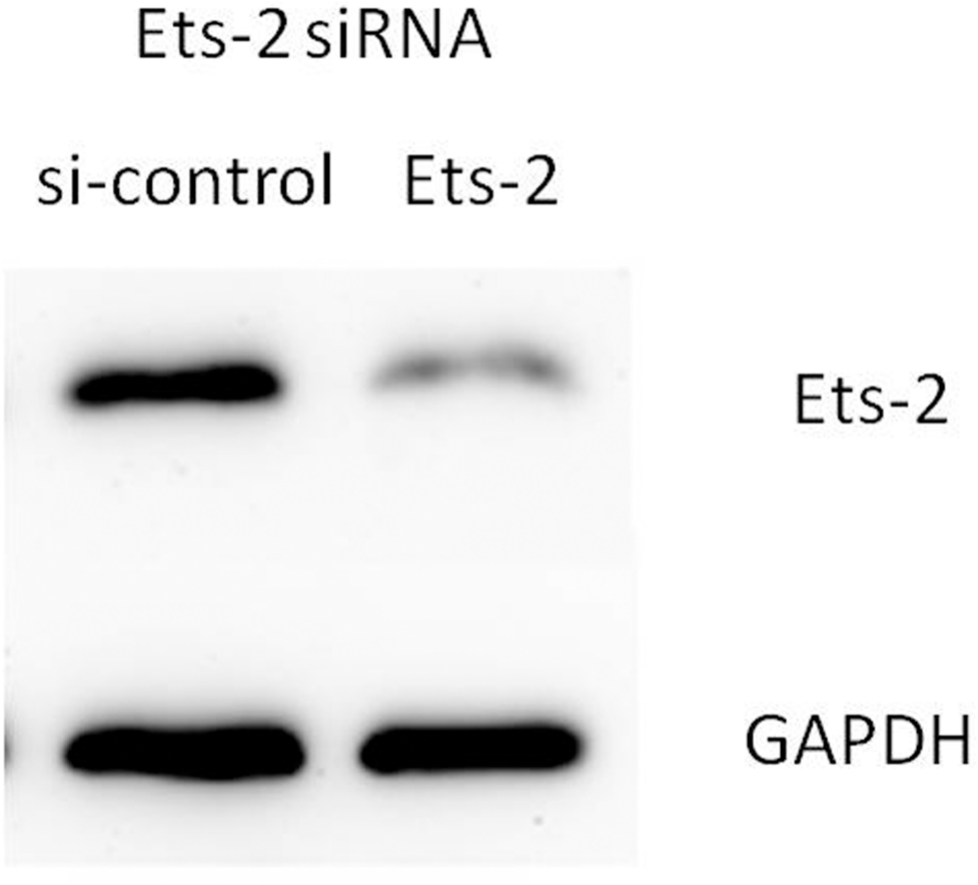
Ets-2 siRNA regulates Ets-2 protein expression in HUVECs. HUVECs were transfected with Ets-2 siRNA for 48h, and the expression of Ets-2 protein levels were detected by western blot assay.

**Fig 12:**
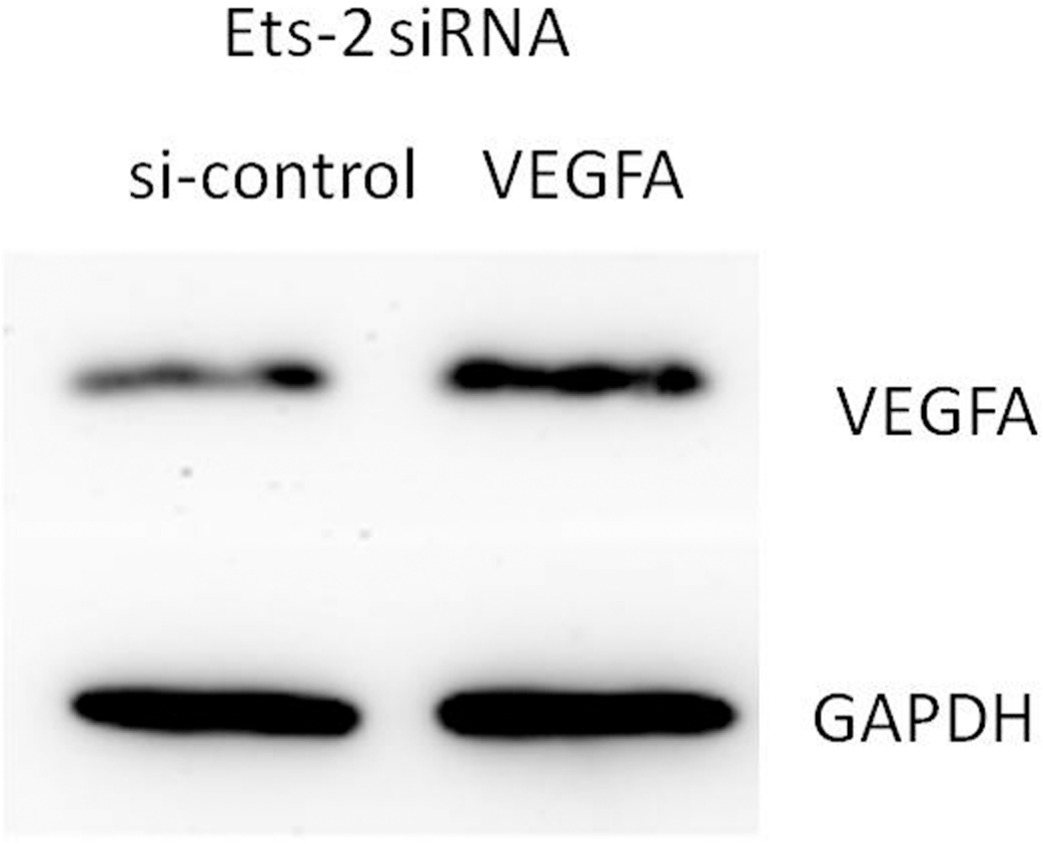
Ets-2 siRNA regulates VEGFA protein expression in HUVECs. HUVECs were transfected with Ets-2 siRNA for 48h, the expression of VEGFA protein levels were detected by western blot assay.

To determine whether Ets-2 regulates VEGFA protein expression, HUVECs were treated with Ets-2 siRNA, and Ets-2 and VEGFA protein levels were analyzed by Western blotting. The results show that Ets-2 siRNA inhibited Ets-2 protein expression and increased VEGFA protein expression (Figs 11 and 12).

### Ets-2 siRNA regulates miR-126 and VEGFA mRNA expression in HUVECs

In this study, we found that ATO upregulates the expression of Ets-2, miR-126, and VEGFA in HUVECs treated with 1.0 μmol/L, 2.5 μmol/L, and 5.0 μmol/L ATO (Figs 5 and 8). We therefore hypothesized that ATO might regulate miR-126 levels by regulating Ets-2.

**Fig 13:**
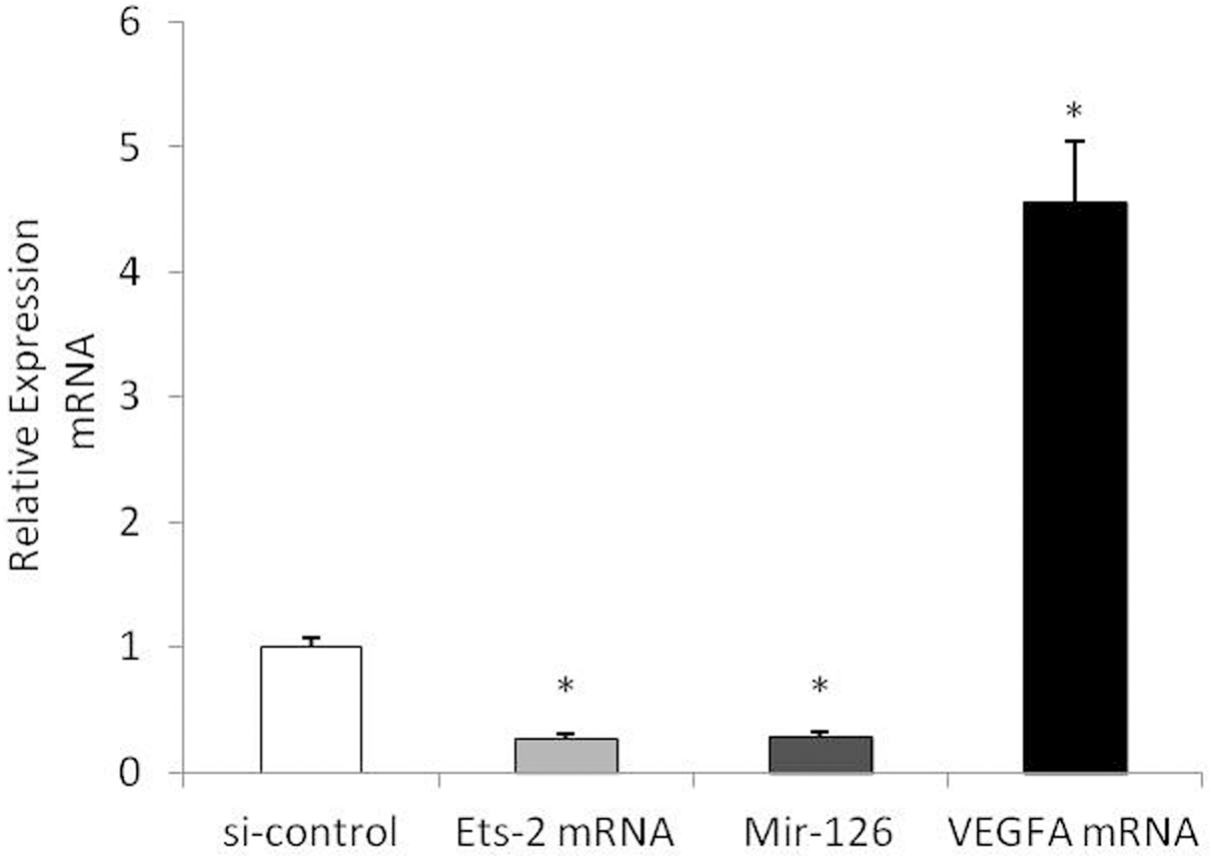
Ets-2 siRNA regulates miR-126 and VEGFA mRNA expression in HUVECs. HUVECs were transfected with Ets-2 siRNA for 48h, the expression of mRNAs of Ets-2, miR-126 and VEGFA mRNA were detected by real-time PCR assay.

To explore the association of Ets-2 with miR-126 and VEGFA, HUVECs were treated with Ets-2 siRNA, and the expression of Ets-2 mRNA, miR-126 and VEGFA mRNA was measured. Ets-2 mRNA and miR-126 expression decreased significantly after transfection with Ets-2 siRNA, whereas VEGFA mRNA expression increased significantly after Ets-2 siRNA transfection (Fig 13). These findings suggested that Ets-2 siRNA upregulated VEGFA expression via miR-126.

### miR-126 upregulation caused by ATO is through Ets-2

**Fig 14:**
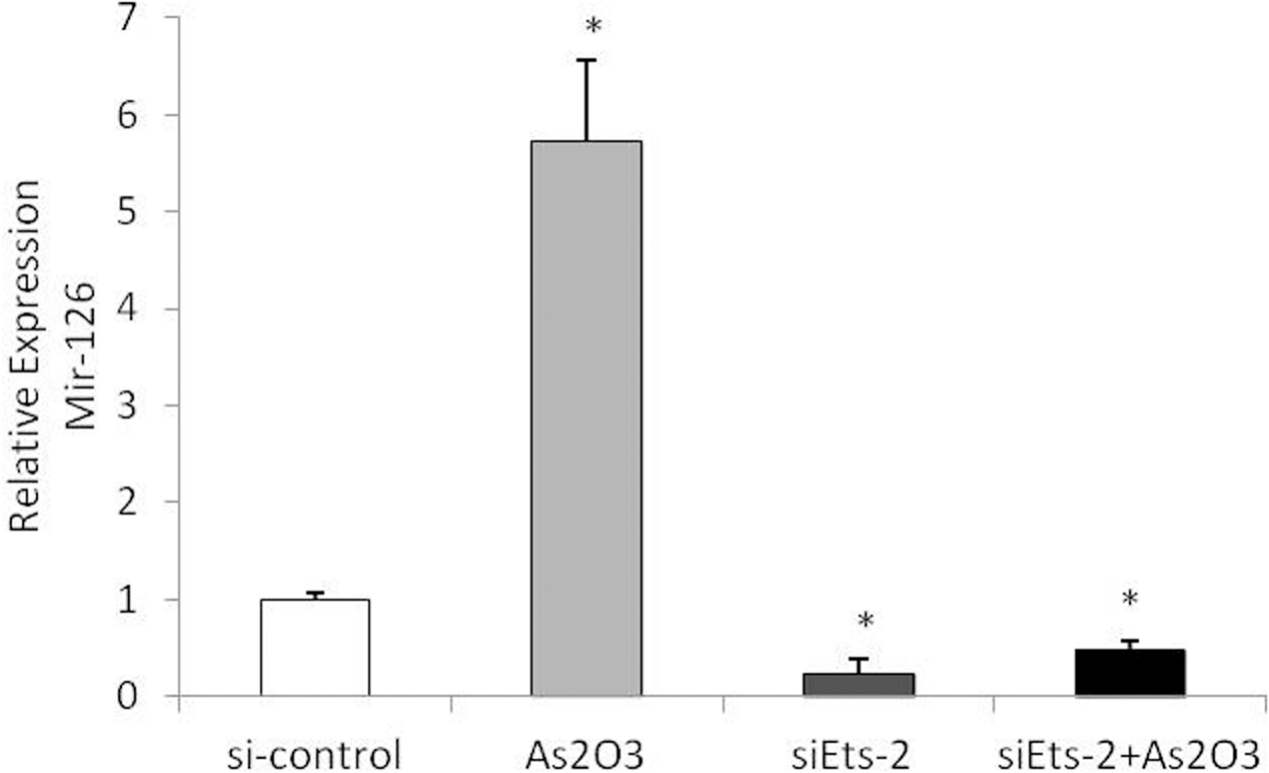
Ets-2 siRNA regulate expression in HUVECs. HUVECs were transfected with Ets-2 siRNA for 6h, subsequently treated with or without high concentration (5.0umol/L)of As_2_O_3_, either 5.0μmol/L ATO or medium alone was added, and the cells were cultured for an additional 48h. The expression of miR-126 was detected by real-time PCR assay.

To determine whether miR-126 upregulation caused by ATO is through Ets-2, HUVECs were treated with Ets-2 siRNA, and then with 5.0 μmol/L ATO. Real-time PCR was used to detect the expression of miR-126. The results show that miR-126 expression was not upregulated by ATO after Ets-2 was knockdown, which indicated that ETS2 up-regulation caused by ATO is inducing miR-126(Fig 14).

### miR-126 is involved in the induction of apoptosis following ATO treatment

To determine whether miR-126 induces apoptosis in HUVECs, we used flow cytometry to investigate the level of apoptosis, and we analyzed the combined effect of ATO (2.5 μmol/L) and either the miR-126 mimic or the miR-126 inhibitor.

**Fig 15:**
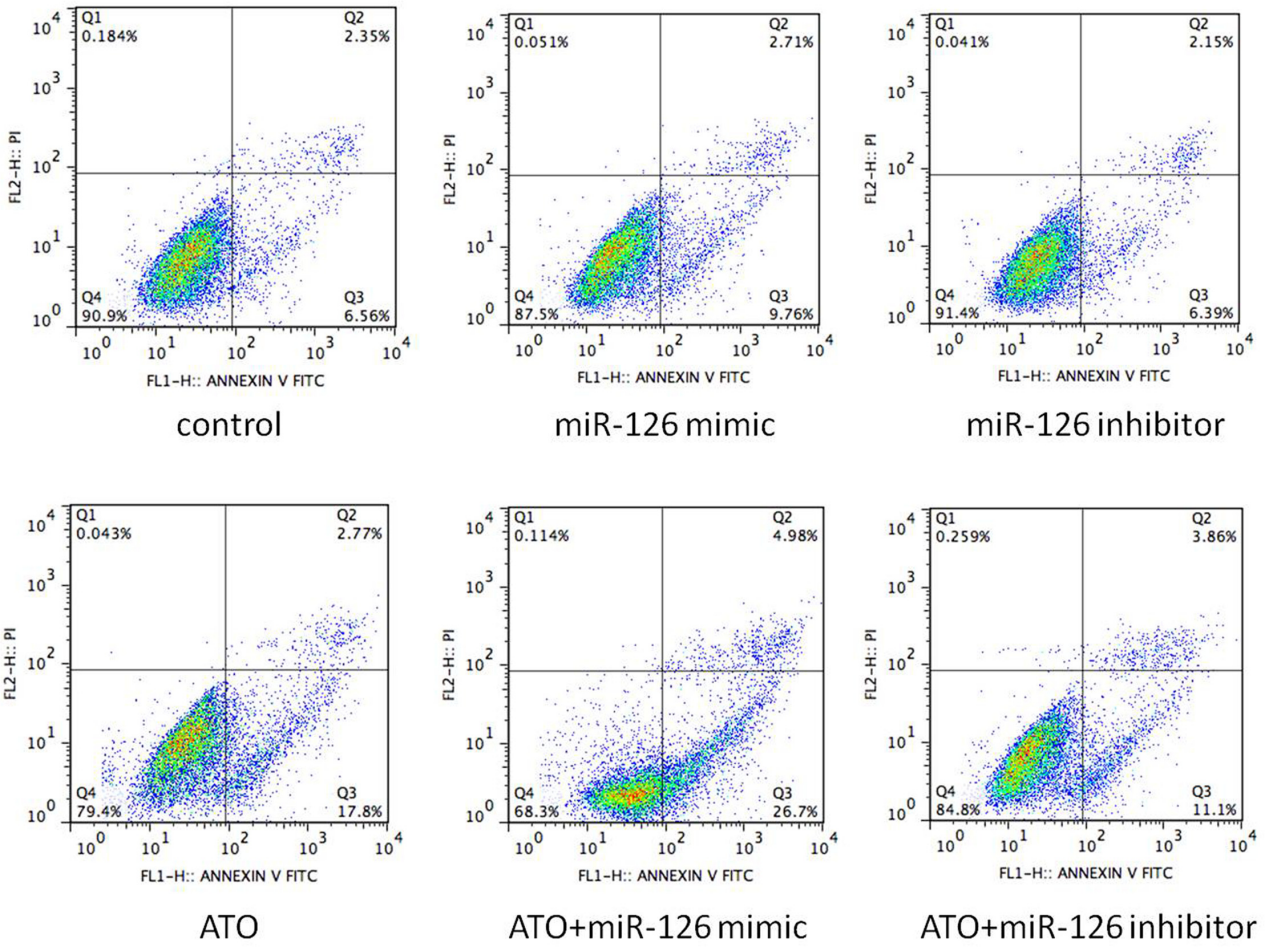
miR-126 is involved in the induction of apoptosis following ATO treatment. HUVECs were transfected or non-transfected with miR-126 mimic and miR-126 inhibitor for 6h and subsequently treated with or without high concentration (5.0umol/L) of As_2_O_3_, respectively. After transfection with the miR-126 mimic and the miR-126 inhibitor, either 5.0 μmol/L ATO or medium alone was added, and the transfected cells were cultured for an additional 48 h. Apoptosis rate was measured using an Annexin V/propidium iodide (PI) flow cytometric assay.

We found that overexpression of the miR-126 mimic led to increases in the rate of early apoptosis in HUVECs (a 9.76% increase in the miR-126 mimic group and a more than 6.56% increase in the control group). However, transfection with the miR-126 inhibitor did not decrease the early apoptotic rate significantly compared with the controls. The cells treated with ATO alone showed a significant increase in the early apoptotic rate (17.8%), and the cells treated with ATO after transfection with the miR-126 mimic showed a greater increase (26.7%) compared with the ATO alone group. The cells treated with ATO after transfection with the miR-126 inhibitor showed a decrease (11.1%) in apoptosis compared with the group treated with ATO alone, suggesting that ATO likely induces apoptosis in a miR-126-dependent manner (Fig 15).

### Analysis of cell morphology with electron microscopy confirmed the induction of apoptosis

**Fig 16:**
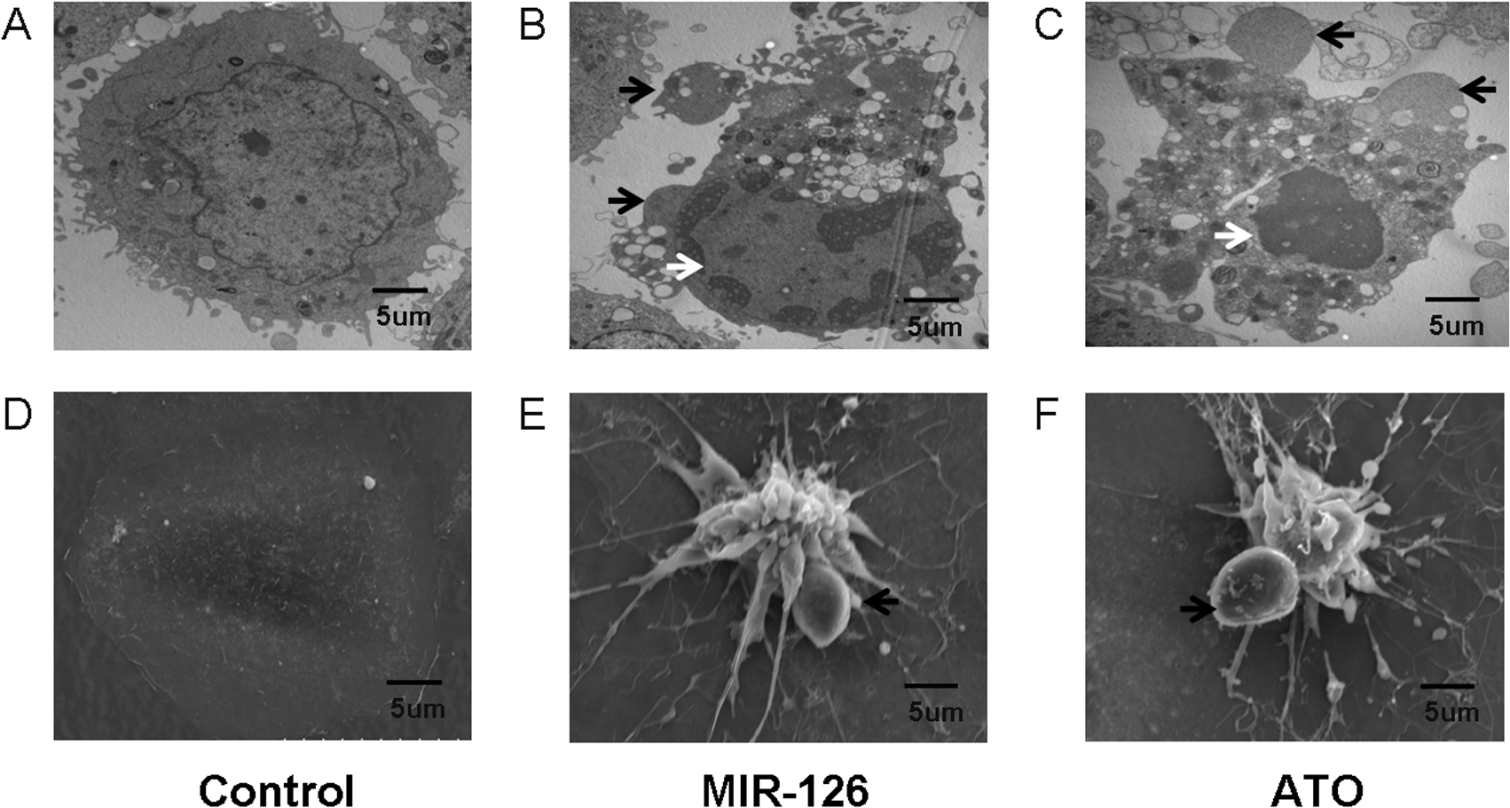
Cell morphology analysis using electron microscopy demonstrates apoptosis. Induction of apoptosis in HUVECs with post-transfection of miR-126 (B and E), ATO(C and F) and control groups(A and D). Cell morphology alteration including cell shrinkage, generated apoptotic body (black arrows) and nucleus pycnosis (white arrows) were shown by electron microscope assay. TEM (A, B and C) and SEM (D, E and F) images of HUVECs were captured at a magnification of 10,000x.

Electron microscopy (SEM and TEM) confirmed that HUVECs transfected with the miR-126 mimic and treated with ATO displayed the typical features of apoptosis, including cell shrinkage, nuclear pyknosis, and apoptotic body formation at 48 h post-transfection. These results demonstrate that ATO is able to induce HUVEC apoptosis via miR-126 upregulation (Fig 16).

## Discussion

Angiogenesis is critical for supporting the growth of tumors and for pathological neovascularization, and angiogenesis blocking methods represent a promising therapeutic approach for the treatment of cancer and neovascularization-related diseases. VEGFA is a potent and specific mitogen for endothelial cells, and it activates the angiogenic switch *in vivo* and enhances vascular permeability; it also plays a key role in the formation of new blood vessels. It has been shown that VEGFA activity is critical for tumor growth and angiogenesis and that blocking this signal transduction pathway can inhibit tumor progression. *In vivo*, VEGFA acts as a potent endothelial cell mitogenic factor and as a blood vessel permeabilizing agent [30]. Recent experiments in mice have illustrated that autocrine VEGFA signaling in endothelial cells is essential for vascular homeostasis [31].

ATO induces apoptosis in leukemic cells and blood vessel endothelial cells in a time- and dose-dependent manner by inhibiting VEGFA production [14]. In recent years, it has been demonstrated that ATO inhibits gastric cancer growth *in vivo* and suppresses the formation of new blood vessels in cancer tissues [16]. In our study, we also found that ATO inhibited the proliferation of HUVECs in a dose-dependent manner. Our data support earlier studies showing that ATO can inhibit VEGFA expression in a dose-dependent manner in HUVECs [14]. The results of the scratch test indicated that ATO (2.5 μmol/L) inhibits the migration of HUVECs after 48 h of treatment.

Several experiments have revealed that the expression of VEGFA is regulated by miR-126 through various target genes; for example, miR-126 influences the repression of PI3KR2 and SPRED1, which are negative regulators of VEGFA signaling, through the MAP kinase and PI3 kinase pathways, respectively [23]. In addition, miR-126 inhibits cell migration and invasion in lung cancer by targeting VEGFA and EGFL-7 [31,32,33].

We used a specific miRNA-126 mimic and a miR-126 inhibitor to study the potential mechanisms of underlying the effects of miR-126 and ATO and to determine the relationship between these treatments in HUVECs. Our data revealed that the miR-126-mimic significantly upregulated mature miRNA-126 expression and downregulated VEGFA mRNA levels. In contrast, transfection with the miR-126 inhibitor downregulated mature miRNA-126 expression and upregulated VEGFA mRNA expression compared to cells transfected with the miR-126 mimic N.C. or the miR-126 inhibitor N.C. The alterations in cellular miRNA and mRNA levels were detected using real-time PCR analysis, and the results indicated that the upregulation of endogenous miR-126 expression contributed to the sensitization of HUVECs to ATO treatment and that this effect was mainly due to an increase in apoptosis.

In our study, we found that ATO downregulated VEGFA protein expression and upregulated ETS-2 mRNA, protein and miR-126 expression in a dose-dependent manner. Our data also showed that ATO upregulated the expression of Ets-2, which induced the expression of miR-126 in endothelial cells, but the level of Ets-1 mRNA did not change significantly.

Harris et al. showed that the transcription factors Ets-1 and Ets-2 regulate miR-126 expression [28]. Based on our data, Ets-2 mRNA and miR-126 expression decreased significantly after Ets-2 siRNA transfection, whereas VEGFA mRNA and protein expression increased significantly after Ets-2 siRNA transfection. At the same time, our results show that miR-126 expression was not upregulated by ATO after Ets-2 was knockdown, which indicated that ETS2 up-regulation caused by ATO is inducing miR-126. These findings suggested that Ets-2 siRNA likely upregulated VEGFA expression via miR-126.

Apoptotic cells were confirmed morphologically using electron microscopy. Flow cytometric assays of cells that were doubly stained with Annexin-V–FITC and PI showed that the proportion of HUVECs in early apoptosis was higher among HUVECs treated with ATO (5.0 μmol/L) than among the control cells. The percentage of cells in early apoptosis in the ATO-treated groups (17.8%) was 2.71 times higher than that observed in the controls (6.56%).

The results also indicated that miR-126 overexpression in HUVECs led to an increase in the rate of early apoptosis and that the rate of early apoptosis was lower after transfection with the miR-126 inhibitor compared with the controls. Similarly, ATO treatment significantly induced early apoptosis in HUVECs, and experiments in which the miR-126 mimic and the miR-126 inhibitor were transfected before treatment with ATO showed that the miR-126 mimic acted synergistically to enhance the effect of ATO, whereas the miR-126 inhibitor counteracted the effect of ATO. Our data suggest that miR-126 might mediate early apoptosis induction in HUVECs by ATO.

In this study, we confirmed that ATO upregulated miR-126. We also found that the combination of miR-126 and ATO induced apoptosis and inhibited growth in HUVECs.

In summary, our findings indicate that ATO inhibits HUVEC proliferation and migration and induces apoptosis in a dose-dependent manner. The negative regulation of VEGFA signaling pathways by ATO might be due to the upregulation of ETS-2 and miR-126. ATO might also act by altering miR-126 expression. The additive action of miR-126 and ATO should be explored due to their potential use in gene therapy for the treatment of tumors and neovascularization-related diseases.
